# The FoxP1 gene regulates lung function, production of matrix metalloproteinases and inflammatory mediators, and viability of lung epithelia

**DOI:** 10.1186/s12931-022-02213-4

**Published:** 2022-10-11

**Authors:** Alexis Andreas, Abby Maloy, Toru Nyunoya, Yingze Zhang, Divay Chandra

**Affiliations:** 1 Department of Medicine, University of Pittsburgh, Pittsburgh, USA; 2 Medical Specialty Service Line, Veterans Affairs Pittsburgh Healthcare System, Pittsburgh, PA USA; 3 Pulmonary, Allergy, and Critical Care Medicine, UPMC Montefiore Hospital-NW628, 3459 Fifth Avenue, Pittsburgh, PA 15213 USA

**Keywords:** COPD, Lung development, FoxP1, IPF, GWAS

## Abstract

**Background:**

Genes involved in lung development may become dysregulated in adult life and contribute to the pathogenesis of lung diseases. Multiple genes regulate lung development, including Forkhead box protein P1-4 (FoxP1-4).

**Methods:**

We examined the association between variants in the FoxP1-4 genes and lung function using data from a GWAS that included close to 400,000 individuals and 20 million SNPs.

**Results:**

More than 100 variants in the FoxP1 gene, but none in the FoxP2-4 genes, are associated with lung function. The sentinel variant in the FoxP1 gene associated with FEV1 was rs1499894 (C > T), while the sentinel variant in the FoxP1 gene associated with FVC was rs35480566 (A > G). Those with the T allele instead of the C allele for rs1499894, or the G allele instead of the A allele for rs35480566 had increased FoxP1 mRNA levels in transcriptomic data, higher FEV1 and FVC, and reduced odds of being diagnosed with idiopathic pulmonary fibrosis. Further, knockdown of FoxP1 in lung epithelial cells by RNA interference led to increased mRNA levels for matrix metalloproteinases 1, 2, 3 and pro-inflammatory cytokines IL-6 & IL-8, as well as reduced cell viability after exposure to cigarette smoke—all processes implicated in the pathogenesis of COPD and IPF.

**Conclusions:**

Our results suggest that the protein encoded by the FoxP1 gene may protect against the development of COPD and IPF. A causal role for FoxP1 in the pathogenesis of COPD and IPF may warrant further investigation, and FoxP1 may be a novel therapeutic target for these lung disorders.

**Supplementary Information:**

The online version contains supplementary material available at 10.1186/s12931-022-02213-4.

## Introduction

Poorly developed lungs are an accepted risk factor for COPD. However, it is not as well appreciated that developmental genes can become dysfunctional in adult life, and contribute to COPD as well as other lung diseases such as IPF [1, 2]. Several genes are critical for proper lung development, including the 4 members of the P sub-family of Forkhead box transcription factors (FoxP1-4) [3, 4]. However, the relationship between variants in the FoxP genes and lung function in adults has not been fully examined.

Variants associated with poor lung function are also associated with the development of COPD and IPF. For example, variants in the FAM13A locus have been associated with both impaired spirometry and COPD [5]. Similarly, there is shared genetic susceptibility between impaired lung function and IPF [6]. Specifically, variants at the DSP locus have been associated not only with reduced FVC and increased FEV1/FVC but also with a diagnosis of IPF [7].

We examined the association between variants in FoxP1-4 genes and lung function using data from the genome-wide association study performed in the UK BioBank and SpiroMeta cohorts. Further, we investigated if the sentinel variants in these genes were associated with a diagnosis of pulmonary fibrosis, or with changes in gene expression using transcriptomic datasets. Finally, we utilized lung epithelial cells to study the impact of knockdown of the gene on the secretion of matrix metalloproteinases and inflammatory cytokines, as well as cell viability.

We hypothesized that multiple variants in the FoxP genes would be associated with altered lung function, gene expression, and that knockdown would impact the production of matrix metalloproteinases and inflammatory cytokines, as well as the viability of lung epithelia.

## Methods

### GWAS for lung function

Data from GWAS performed using combined data from the UK biobank and SpiroMeta Consortium was analyzed. Details of this GWAS have been described previously by others [8]. In brief, individuals were included in the analysis from the UK Biobank cohort if they (1) had complete data for age, sex, height, and smoking status; (2) had spirometry meeting quality control requirements; (3) had genome-wide imputed data and (4) were of European ancestry. European ancestry was determined based on K means clustering using the first 2 of the 10 principal components provided in the UK Biobank genetics data. 6 clusters were identified including one of European ancestry. Genotyping was undertaken using the Affymetrix Axiom UK BiLEVE and UK Biobank arrays. In total, 321,047 individuals were included from the UK BioBank. Residuals from linear regression of each trait (FEV1, FVC, and FEV1/FVC) against age, age^2^, sex, height, smoking status (ever or never), and genotyping array were ranked and inverse-normal transformed, giving normally distributed Z-scores. These Z-scores were used for GWAS via an additive genetic model using BOLT-LMM v2.3.

The SpiroMeta consortium comprised 79,055 individuals from 22 studies [8]. In each study, linear regression models were fitted for each trait (FEV1, FEV1/FVC, and FVC), with adjustment for age, age^2^, sex, and height.

A total of 19,819,130 variants (imputed or genotyped) in both UK Biobank and SpiroMeta were meta-analyzed, using inverse-variance-weighted fixed-effect meta-analysis as described previously [8]. The sentinel variant was defined as the variant in an association signal (*p* < 5 × 10^−9^) where no other variants within 1 Mb showed a stronger association. For conditional analysis, SNPs ± 1 Mb from the sentinel variants were extracted and used for stepwise conditional analysis to select independently associated SNPs within each 2-Mb region, using GCTA. Results were summarized as Manhattan plots and regional association plots.

Linkage disequilibrium was assessed using LDlink and is reported as the *r*^*2*^ [9].

### GWAS for IPF

Genetic signals for impaired lung function often overlap with those for fibrotic lung disorders, the most common of which is idiopathic pulmonary fibrosis (IPF). Therefore, after identifying sentinel SNPs associated with lung function, we determined if these variants were also associated with a diagnosis of IPF. We did this using previously published GWAS data that combined data from 3 studies (n = 2668 cases and 8591 controls with 10,790,934 well-imputed variants) [7].

### eQTL analysis

Most single nucleotide polymorphisms (SNPs) are in non-coding/intronic regions and therefore do not alter the sequence of encoded proteins. Even though these SNPs are in intronic regions they may exert biologically significant effects by regulating the expression of nearby genes. Such SNPs are called eQTLs or expression quantitative trait loci.

We determined if sentinel SNPs in the FoxP genes were eQTLs using the GTex and QTLbase datasets [10]. Results are summarized as violin plots.

### Cell culture and RNA interference

BEAS-2B cells were obtained from ATCC (Manassas, VA) and cultured in Hite’s Medium (DMEM F12 with 10% fetal bovine serum, 2 mM L-glutamine, 1 × non-essential amino acids, 10 mM HEPES, 1 mM sodium pyruvate, and 1 × Penicillin/Streptomycin) in 10 cm dishes. Cells were transfected with 50 nM dicer substrate small interfering RNA (dsi RNA, Integrated DNA Technologies, Coralville, Iowa) using GenMute transfection reagent (Signagen, Frederick, MD) once 60–70% confluent.

## RT-PCR

RNA was isolated from cells using RNeasy Mini Kits (Qiagen) per the manufacturer’s instructions. Isolated RNA were immediately converted to cDNA using High Capacity RNA-to-cDNA Kits (Life Technologies, Grand Island, NY) after their concentrations were measured. Real-time PCR assays were performed using SYBR® Select Master Mix for CFX (2X) (Life Technologies, Grand Island, NY) with the C1000 Thermal Cycler (BioRad, Hercules, CA) per manufacturer instructions. Primer sequences are listed in supplementary materials.

### Immunoblotting

Supernatants were discarded and the cells were washed twice with ice-cold PBS. Lysis buffer was added (0.5% Triton in PBS with 1 × Halt protease inhibitor cocktail) and the lysate was mechanically scraped off the bottom of each well. Lysates were sonicated for 15 s on ice, protein concentration measured (DC protein assay, Bio-Rad), and 20 μg/lane of lysate loaded onto a 10% stain-free SDS-PAGE gel (Bio-Rad). After electrophoresis, the gel was photographed under UV light to image the protein in the gel according to manufacturer instructions. After transfer to a low fluorescence PVDF membrane using a Trans-Blot Turbo (Bio-Rad, Hercules, CA) the membrane was re-imaged under UV light to confirm complete and even transfer of the protein from the gel. The membrane was then blocked with 3% (w/v) BSA in TBST (25 mM Tris–HCl, pH 7.4, 137 mM NaCl, and 0.1% Tween 20) for 1 h, and incubated with primary antibody in 3% (w/v) BSA in TBST at 4° C overnight or 1–2 h at RT. The membrane was then washed 3 times with TBST at 5-min intervals followed by a 1 h incubation with horseradish peroxidase-conjugated secondary antibody (1:2000). The membrane was developed with an enhanced chemiluminescence detection system according to the manufacturer's instructions. Densitometry was performed and adjusted to total protein per lane using automated measurement and rolling disk normalization using Image Lab v5.2.1 according to manufacturer instruction (Bio-Rad).

### Cell viability

Cells were detached using 0.25% Trypsin–EDTA and washed three times with PBS. They were then stained with a vital dye per manufacturer instructions (LIVE/DEAD™ Fixable Aqua Dead Cell Stain Kit, Thermo Fisher). Cells were then fixed and subject to flow using a BD LSR Fortessa flow cytometer (San Jose, CA) to capture > 10,000 events/sample. Results were analyzed using FlowJo (Ashland, OR). Cells were identified by plotting forward scatter area against side scatter area (FSC-A/SSC-A gate), and doublets were excluded by plotting forward scatter area against forward scatter height (FSC-A/FSC-H gate). The threshold for classifying cells as live or dead was a signal intensity > 10^3^ at 405 nm wavelength.

### Measurement of matrix metalloproteinases and pro-inflammatory cytokines

Matrix metalloproteinase (MMP) 1, 2, 3 along with IL-6 and IL-8 levels were assessed in the supernatant using ELISA per manufacturer instructions (Ray Biotech, Peachtree Corners, GA). These analytes were chosen given their established role in matrix turnover and inflammation in chronic lung diseases such as COPD [11–14]. We did not assess levels for other MMP such as MMP 7, 9, and 12 because they are not expressed by BEAS-2B cells per our prior RNA sequencing results.

### Preparation of cigarette smoke extract (CSE)

Research cigarettes (1R6F) were purchased from the University of Kentucky (Lexington, Kentucky, USA). CSE was prepared using the method of Blue and Janoff [15]. Through one opening of a stopcock, 10 ml of sterile DMEM was drawn into a 50-ml plastic syringe. Subsequently, 40 ml of cigarette smoke was drawn into the syringe and mixed with the medium by vigorous shaking. One cigarette was used for every 10 ml of medium. The generated CSE solution was filtered (0.22 μm). The resulting solution was designated a 100% CSE solution. The CSE solution was used within 10 min.

### Statistical analysis

Descriptive statistics were reported as median, IQR. Non-parametric methods were used unless stated otherwise. Analyses were performed using STATA v17 (StataCorp, College Station, TX), R v4.1.1, and GraphPad Prism v7.0 (GraphPad Software, La Jolla, CA). A two-tailed *p* < 0.05 was considered statistically significant.

## Results

### rs1499894 and rs35480566 are the sentinel variants in the FoxP1 gene that are associated with FEV1 and FVC, respectively

**Fig. 1:**
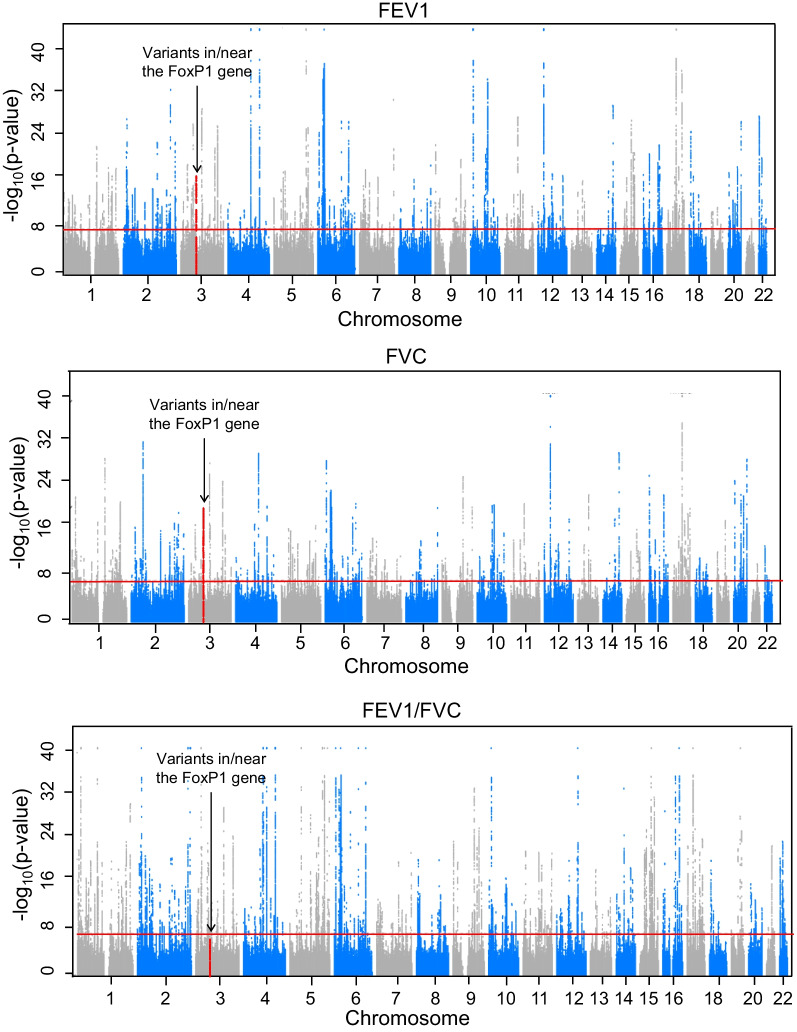
Variants in/near the FoxP1 gene are associated with lung function in combined data from the UK Biobank and SpiroMeta cohorts. Manhattan plots summarizing the association between variants in the genome and lung function. Loci crossing the red line were statistically significant at the genome-wide level

**Fig. 2:**
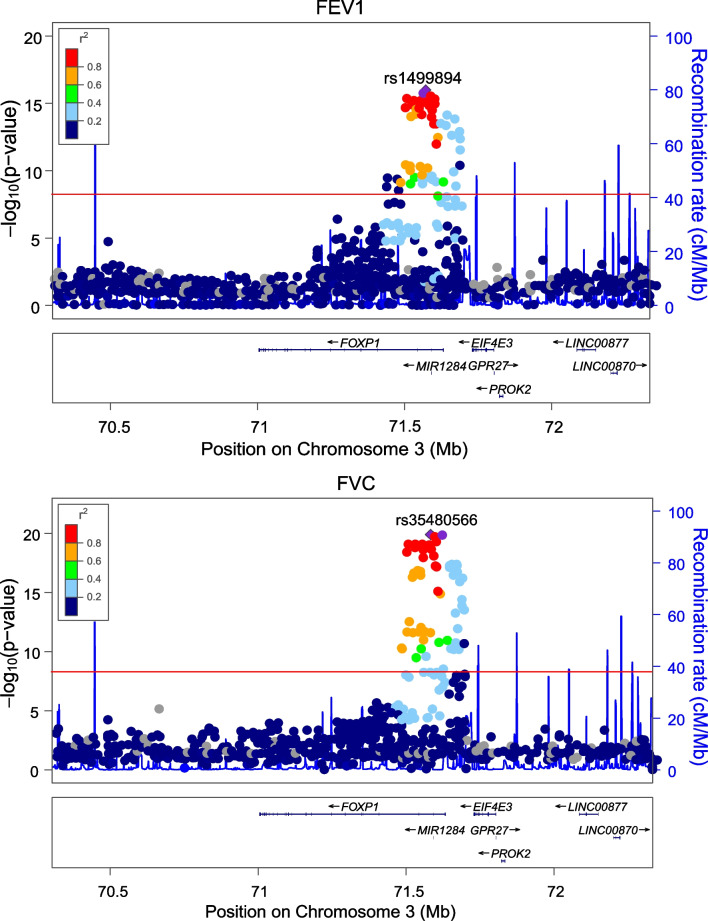
Multiple SNPs in the FoxP1 gene are associated with lung function in the combined data from the UK Biobank and SpiroMeta cohorts. Regional association plots summarizing the association between variants in the region of chromosome 3 where the FoxP1 gene is located and measures of lung function. Variants located above the redline were statistically significant at the genome-wide level. 135 SNPs in the FoxP1 gene had a statistically significant association with FEV1 while 121 had a statistically significant association with FVC

In the combined UK Biobank and SpiroMeta data, multiple variants in the FoxP1 gene were genome-wide significant for FEV1 and FVC, but none were significant for FEV1/FVC (Fig. 1). Regional association plots demonstrated that 135 FoxP1 SNPs were genome-wide significant for FEV1 while 121 were genome-wide significant with FVC (Fig. 2). 119 of these SNPs were significant for both FEV1 and FVC, while 2 were statistically significant for FVC only, and 16 were statistically significant for FEV1 only. The sentinel SNP for FEV1 was rs1499894 and for FVC was rs35480566. Further adjustment for BMI had no impact. Specifically, we found no statistically significant association between the FoxP1 SNPs that were genome-wide significant for FEV1 or FVC with BMI, and further adjustment for BMI had no impact on the association of these variants with lung function.

The two sentinel variants (rs1499894 and rs35480566) were in high linkage disequilibrium with each other (*r*^*2*^ = 0.935). Therefore, the effect estimates for the two variants were almost identical as these variants represent essentially the same information (Table 1). Conditional analysis did not reveal the presence of more than one association signal at the FoxP1 locus.

**Table 1:** The association of the sentinel variants in the FoxP1 gene with FEV1 and FVC

SNP			Meta-analysis (FVC)			Meta-analysis (FEV1)		
	Major allele	Minor allele	β*	*p*	Direction†	β*	*p*	Direction†
rs35480566	C	T	0.022	1.25E−20	+ / +	0.019	1.79e−16	+ / +
rs1499894	A	G	0.022	1.82E−20	+ / +	0.019	1.02e−16	+ / +

*The β represents the change in the Z-score for the spirometry variable

†The direction of the association in the two cohorts (UK BioBank/SpiroMeta) separately

In contrast, no variant in the FoxP2, FoxP3, or FoxP4 gene had a statistically significant association with FEV1, FVC, or FEV1/FVC. Therefore, we focused our attention on the sentinel variants in the FoxP1 gene for further investigation.

### Sentinel SNPs in the FoxP1 gene are associated with the odds of being diagnosed with IPF

Analysis of an unrelated GWAS study on IPF suggested that rs1499894 and rs35480566 were associated with the odds of being diagnosed with IPF (Table 2).

**Table 2:** Sentinel SNPs in the FoxP1 gene that were associated with better lung function in the UK BioBank were also associated with odds of being diagnosed of IPF in an independent GWAS study

SNP	Chromosome	Position	Major allele	Minor allele	OR	*p*
rs1499894	3	71571696	C	T	0.93 (0.87–0.99)	0.03
rs35480566	3	71583177	A	G	0.92 (0.86–0.98)	0.02

### Sentinel SNPs in the FoxP1 gene were eQTLs for FoxP1 in peripheral blood and EIF4E3 in prostate tissue

**Fig. 3:**
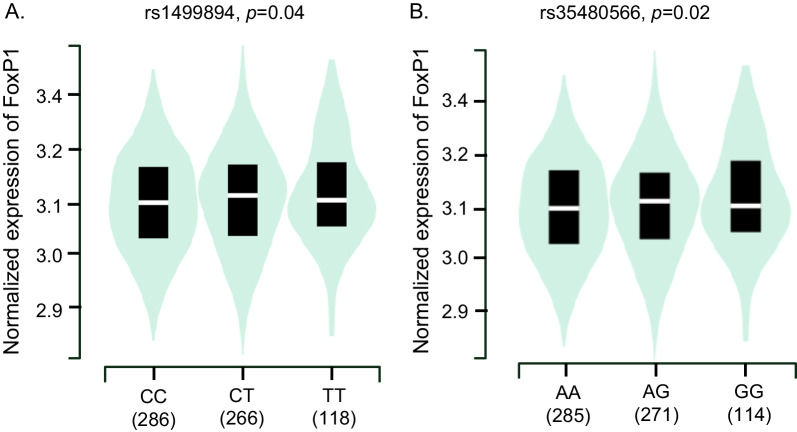
The sentinel SNPs in the FoxP1 gene were also eQTLs, i.e., individuals who carry these variants have higher levels of FoxP1 mRNA in peripheral blood (**A**, **B**)

Carriers of either rs1499894 or rs35480566 had higher FoxP1 mRNA levels in peripheral blood (Fig. 3, panel A and B). Also, carriers of rs1499894 had altered levels of the mRNA expressed by a gene in the vicinity of the FoxP1 gene on chromosome 3 i.e., EIF4E3 in prostate tissue (Additional file 1: Fig. S1, panel A).

Because rs1499894 was associated with altered mRNA levels for FoxP1 and EIF4E3 we hypothesize that FoxP1 and EIF4E3 are biologically inter-related [16]. To test this hypothesis, we depleted FoxP1 mRNA in lung epithelial cells (BEAS-2B) by RNAi. We chose lung epithelial cells because others have demonstrated by immunohistochemical staining that FoxP1 protein is primarily present in the epithelial compartment of adult lungs [3]. We confirmed ~ 75% knockdown of both FoxP1 mRNA and protein levels (Additional file 1: Fig. S1, panel B–D). Loss of FoxP1 altered EIF4E3 mRNA levels assessed by RT-PCR (Additional file 1: Fig. S1, panel E). EIF4E3 belongs to the EIF4E family of translation initiation factors that interact with the 5-prime cap of mRNA and recruit mRNA to the ribosome [17]. Members of this family of genes have been implicated in cell proliferation, organ development, regulation of protein synthesis, and carcinogenesis [18–20].

Overall, these data suggested that loss of FoxP1 may contribute to the development of chronic lung diseases because variants in the FoxP1 gene that were associated with higher mRNA levels were also associated with improved lung function and reduced odds of a diagnosis of IPF.

### Knockdown of FoxP1 leads to increased secretion of MMPs and inflammatory cytokines by lung epithelia and reduction in cell viability after exposure to cigarette smoke extract

 To test the hypothesis that reduced FoxP1 mRNA levels potentiate tobacco-related lung diseases such as COPD and IPF, we measured levels of MMP 1, 2, 3, and IL 6 and 8, as well as cell viability in lung epithelial cells with and without FoxP1 knockdown by RNAi.

**Fig. 4:**
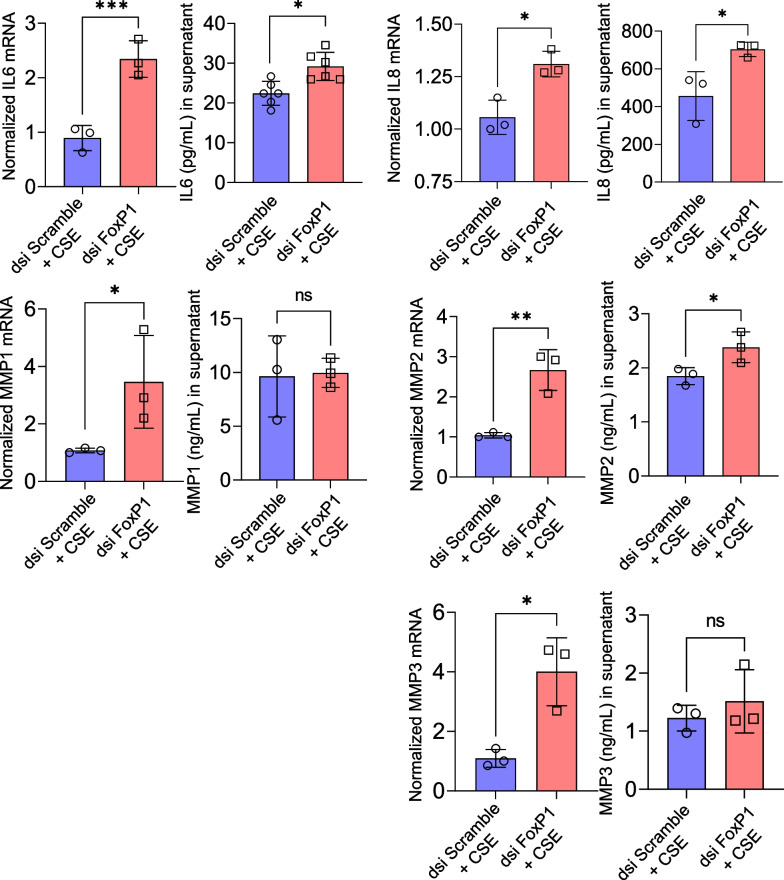
Depletion of FoxP1 by RNA interference increased intracellular mRNA levels and protein levels in the supernatant of inflammatory mediators and matrix metalloproteinases in lung epithelial cells exposed to cigarette smoke extract. Lung epithelial cells were transfected with dsi RNA targeting FoxP1 (dsi FoxP1) or non-sense RNA (dsi Scramble). Cells were then exposed to 5% cigarette smoke extract overnight. Lysate was processed for RT-PCR and supernatant was collected for ELISA. Result are from 3–-6 samples from independent experiments. Non-parametric tests were used (Mann–Whitney U). *p<0.05, **p<0.01, ***p<0.001

Results suggested that loss of FoxP1 led to increased mRNA for all five mediators in cells exposed to cigarette smoke extract (Fig. 4). The levels of most of these mediators was also increased in the supernatant (Fig. 4). In cells that had not been exposed to cigarette smoke extract, changes in mRNA levels for the 5 mediators were less impressive (Additional file 1: Fig S2). Accordingly, levels of these mediators in the supernatant were not assessed in cells not exposed to cigarette smoke extract.

**Fig. 5:**
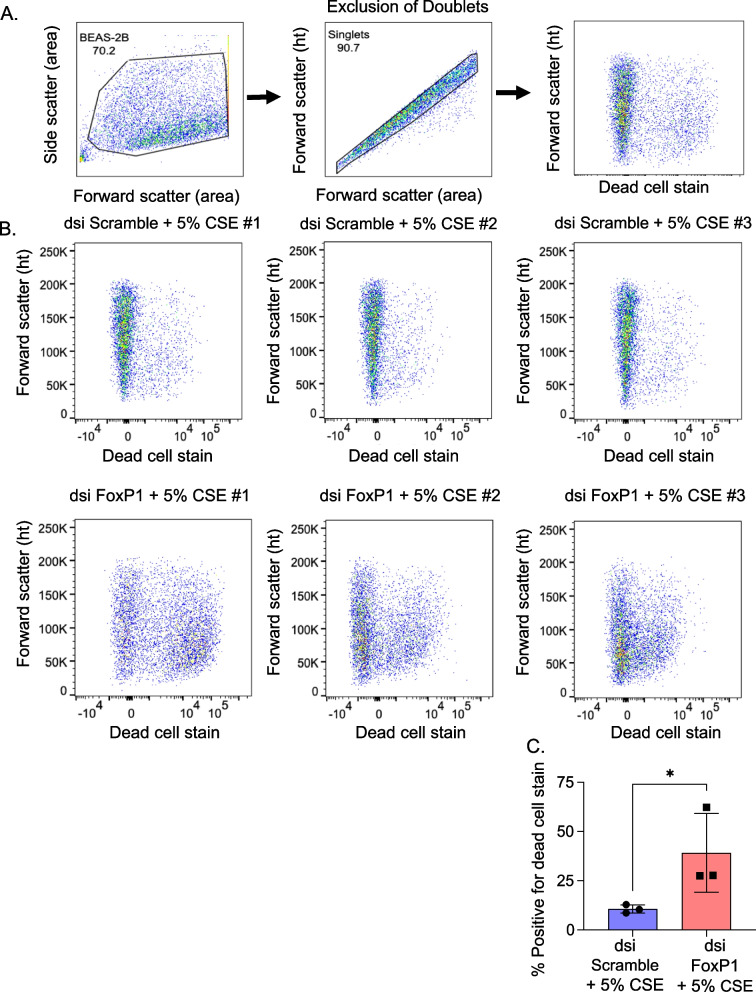
Depleting FoxP1 by RNA interference reduced the viability of lung epithelial cells exposed to cigarette smoke extract. Lung epithelial cells were transfected with dsi RNA targeting FoxP1 mRNA (dsi FoxP1) or non-sense mRNA (dsi Scramble). 24h later cells were exposed to 5% cigarette smoke extract overnight and then stained with Zombie Aqua blue (stains non-viable cells). Flow cytometry was performed (gating strategy per **A**) on 3 biological replicates to record 10,000 events per sample. Cells transfected with dsi FoxP1 showed lower viability than cells transfected with dsi Scramble (**B**). The differences in % of dead cells were statistically significant (**C**). Non-parametric tests were used (Mann–Whitney U). *p<0.05

Results also suggested that loss of FoxP1 leads to greatly reduced cell survival. Specifically, the % positivity for the dead cell stain increased ~ 50% with knockdown of FoxP1 versus control knockdown in cells exposed to cigarette smoke extract (Fig. 5). A similar 50% increase was identified in cells that had not been exposed to cigarette smoke extract (Additional file 1: Fig S3).

These data suggest that the protein encoded by the FoxP1 gene may be an important regulator of lung epithelial inflammation, matrix turnover, and cell survival and, therefore, may warrant further investigation.

## Discussion

Genes involved in lung development may play a critical role in the pathogenesis of lung disease in adult life. Lung development is regulated by a number of genes, including Forkhead box protein P1-4 (FoxP1-4) [3, 21]. Therefore, we examined the association between variants in the FoxP genes and lung function. Results suggest that more than 100 variants in the FoxP1 gene are associated with changes in lung function, while no variants in FoxP2-4 were statistically significant. The sentinel variants in the FoxP1 gene are associated with increased FoxP1 mRNA levels, higher FEV1 and FVC, and reduced odds of IPF. Further, knockdown of FoxP1 in lung epithelial cells led to increased secretion of matrix metalloproteinases and inflammatory cytokines after exposure to cigarette smoke extract. Also, knockdown of FoxP1 reduced viability of lung epithelial cells. These findings suggest that FoxP1 may have a protective role against tobacco-related lung diseases such as COPD and IPF and warrant further investigation.

The Forkhead box (Fox) family includes 50 transcription factors in subclasses A-S characterized by a forkhead box DNA binding domain. These proteins are conserved from yeast to humans and regulate diverse biological processes during development and adult life [22, 23]. Four subclass P proteins exist in mammals (FoxP1-4) and are important for organ development [3, 4, 21], including that of the lung. Because these transcription factors can heterodimerize with each other they have some degree of functional redundancy [24].

FoxP1 has two known functions in the adult lung: (1) cooperate with FoxP4 to suppress the promoter for protein disulfide isomerase anterior gradient 2 (AGR2) that regulates goblet cell development in the lung epithelium; [21] and (2) suppress the IL-6 promotor in the lung epithelium [25]. However, FoxP1 has no proven role in COPD pathogenesis.

Herein, we implicate the FoxP1 gene as a potential contributor to lung disease in adults. We examined the association of variants in the FoxP1 gene with lung function and a diagnosis of IPF using GWAS data. We then identified that the sentinel variants were associated with higher levels of FoxP1 mRNA. To corroborate these findings, we performed functional studies in lung epithelial cells where loss of FoxP1 mRNA was associated with adverse effects such as increased production of MMPs and IL-6 & 8 as well as reduced cell viability. Therefore, our findings implicate the FoxP1 gene as a protective mediator against chronic lung diseases such as COPD and IPF. Further investigation of the role of FoxP1 in the pathogenesis of COPD may be warranted.

Although the FoxP1-4 genes have been implicated in lung development, we were able to associate variants in only the FoxP1 gene with lung function. This finding suggests that FoxP1 may have a unique role in regulating lung function. However, it is also possible that there was less coverage of variants important for regulating lung function in the FoxP2-4 genes vs. the FoxP1 gene in the genotyping arrays.

Our study corroborates a prior report where FoxP1 protein repressed the promoter for the IL-6 gene [25]. Specifically, we showed that when FoxP1 protein was knocked down, IL-6 levels in the supernatant increased, although only in the presence of cigarette smoke extract. It is likely that cigarette smoke extract increased activation of inflammatory pathways and thus exacerbated differences in IL-6 levels between dsi FoxP1 and control knockdown. Because FoxP1 protein is a transcriptional repressor, one can speculate that FoxP1 also represses the promoters of IL-8, MMPs, and EIF4E3 although we were unable to locate any publicly available FoxP1 ChIP-seq data in lung cells to confirm this hypothesis. Other mechanisms may also be responsible. For example, FoxP1 may regulate mediators that are upstream of IL-8, MMPs, and EIF4E3. Also, there may be crosstalk between inflammatory and matrix turnover pathways. The responsible mechanisms need to be elucidated in vitro and in vivo.

Our study has limitations. Although we have reported associations between variants in the FoxP1 gene and FoxP1 mRNA levels as well as functional effects in vitro, we have not proven the mechanism or demonstrated a causal role for FoxP1 in the pathogenesis of COPD or IPF. Second, our data suggest that FoxP1 may play a role in the development of lung diseases in adults caused by tobacco exposure. However, we were unable to determine if FoxP1 is associated with poor lung function in adults because of poor lung development. Also, the association between sentinel variants in the FoxP1 gene and diagnosis of IPF was nominally significant (*p* < 0.05) rather than genome-wide significant. We suspect, but cannot prove, that this was due to the much smaller sample size, and therefore reduced statistical power, of IPF GWAS studies vs. the lung function GWAS in the UK Biobank. Finally, the GWAS model was not further adjusted for pack-years of smoking by us because the majority of participants were never smokers, and pack-year data was not available on a large proportion of ever smokers (~ 30%).

## Conclusions

We examined the association between variants in the FoxP1-4 genes and lung function in adults. Results suggest that more than 100 variants in the FoxP1 gene are associated with changes in lung function. In contrast, no variants in the FoxP2-4 are associated with lung function. The sentinel variants in the FoxP1 gene are associated with increased FoxP1 mRNA levels, higher FEV1 and FVC, and reduced odds of IPF. Further, knockdown of FoxP1 in lung epithelial cells had deleterious consequences such as increased secretion of matrix metalloproteinases and inflammatory cytokines as well as increased cell death. Our results suggest that the FoxP1 gene may be protective against the development of COPD and IPF. A causal role for FoxP1 in the pathogenesis of COPD and IPF may warrant further investigation, and FoxP1 may be a potential novel therapeutic target for these lung disorders.

## Supplementary Information

**Additional file 1: Figure S1.** rs1499894 was associated with altered levels of EIF4E3 mRNA in prostate tissue (A). To validate the association between FoxP1 and EIF4E3 mRNA levels, we depleted FoxP1 mRNA in lung epithelial cells by RNAi (dsi FoxP1) and confirmed altered EIF4E3 mRNA levels relative to dsi control (B-E). Non-parametric tests (Mann-Whitney U) were used to calculate p-values from 3-6 samples from independent experiments. **** p<0.001, * p<0.05. **Figure S2.** Depletion of FoxP1 by RNA interference did not significantly alter mRNA levels of inflammatory mediators and matrix metalloproteinases in lung epithelial cells in the absence of cigarette smoke extract. Lung epithelial cells were transfected with dsi RNA targeting FoxP1 (dsi FoxP1) or non-sense RNA (dsi Scramble). 48h later lysate was processed for RT-PCR. Cells transfected with dsi FoxP1 did not show greater mRNA levels for IL6, IL8, MMP1, MMP2, and MMP3. Non-parametric tests were used (Mann-Whitney U). *ns = not significant. **Figure S3.** Depleting FoxP1 by RNA interference did not reduce the viability of lung epithelial cells in the absence of cigarette smoke extract. Lung epithelial cells were transfected with dsi RNA targeting FoxP1 mRNA (dsi FoxP1) or non-sense mRNA (dsi Scramble). 48h later cells were stained with Zombie Aqua blue (stains non-viable cells). Flow cytometry was performed (gating strategy per A) on 3 biological replicates to record 10,000 events per sample. Cells transfected with dsi FoxP1 showed similar viability to cells transfected with dsi Scramble (B). Non-parametric tests were used (Mann-Whitney U).

## Data Availability

SpiroMeta GWAS summary statistics and UK Biobank GWAS summary statistics are available online via LD-Hub (http://ldsc.broadinstitute.org/ldhub/). The newly derived spirometry variables are available from UK Biobank (http://www.ukbiobank.ac.uk/). Data from in vitro experiments are available from the authors on request.
