# Supplementary material for: The FoxP1 gene regulates lung function, production of matrix metalloproteinases and inflammatory mediators, and viability of lung epithelia
Source: Respir Res. 2022 Oct 11;23:281. doi: 10.1186/s12931-022-02213-4 (PMC9554985; doi:10.1186/s12931-022-02213-4)
Supplement: Supplementary file 1 — Additional file 1: Figure S1. rs1499894 was associated with altered levels of EIF4E3 mRNA in prostate tissue (A). To validate the association between FoxP1 and EIF4E3 mRNA levels, we depleted FoxP1 mRNA in lung epithelial cells by RNAi (dsi FoxP1) and confirmed altered EIF4E3 mRNA levels relative to dsi control (B-E). Non-parametric tests (Mann-Whitney U) were used to calculate p-values from 3-6 samples from independent experiments. **** p<0.001, * p<0.05. Figure S2. Depletion of FoxP1 by RNA interference did not significantly alter mRNA levels of inflammatory mediators and matrix metalloproteinases in lung epithelial cells in the absence of cigarette smoke extract. Lung epithelial cells were transfected with dsi RNA targeting FoxP1 (dsi FoxP1) or non-sense RNA (dsi Scramble). 48h later lysate was processed for RT-PCR. Cells transfected with dsi FoxP1 did not show greater mRNA levels for IL6, IL8, MMP1, MMP2, and MMP3. Non-parametric tests were used (Mann-Whitney U). *ns = not significant. Figure S3. Depleting FoxP1 by RNA interference did not reduce the viability of lung epithelial cells in the absence of cigarette smoke extract. Lung epithelial cells were transfected with dsi RNA targeting FoxP1 mRNA (dsi FoxP1) or non-sense mRNA (dsi Scramble). 48h later cells were stained with Zombie Aqua blue (stains non-viable cells). Flow cytometry was performed (gating strategy per A) on 3 biological replicates to record 10,000 events per sample. Cells transfected with dsi FoxP1 showed similar viability to cells transfected with dsi Scramble (B). Non-parametric tests were used (Mann-Whitney U). [file 12931_2022_2213_MOESM1_ESM.pptx]

## Slide 1
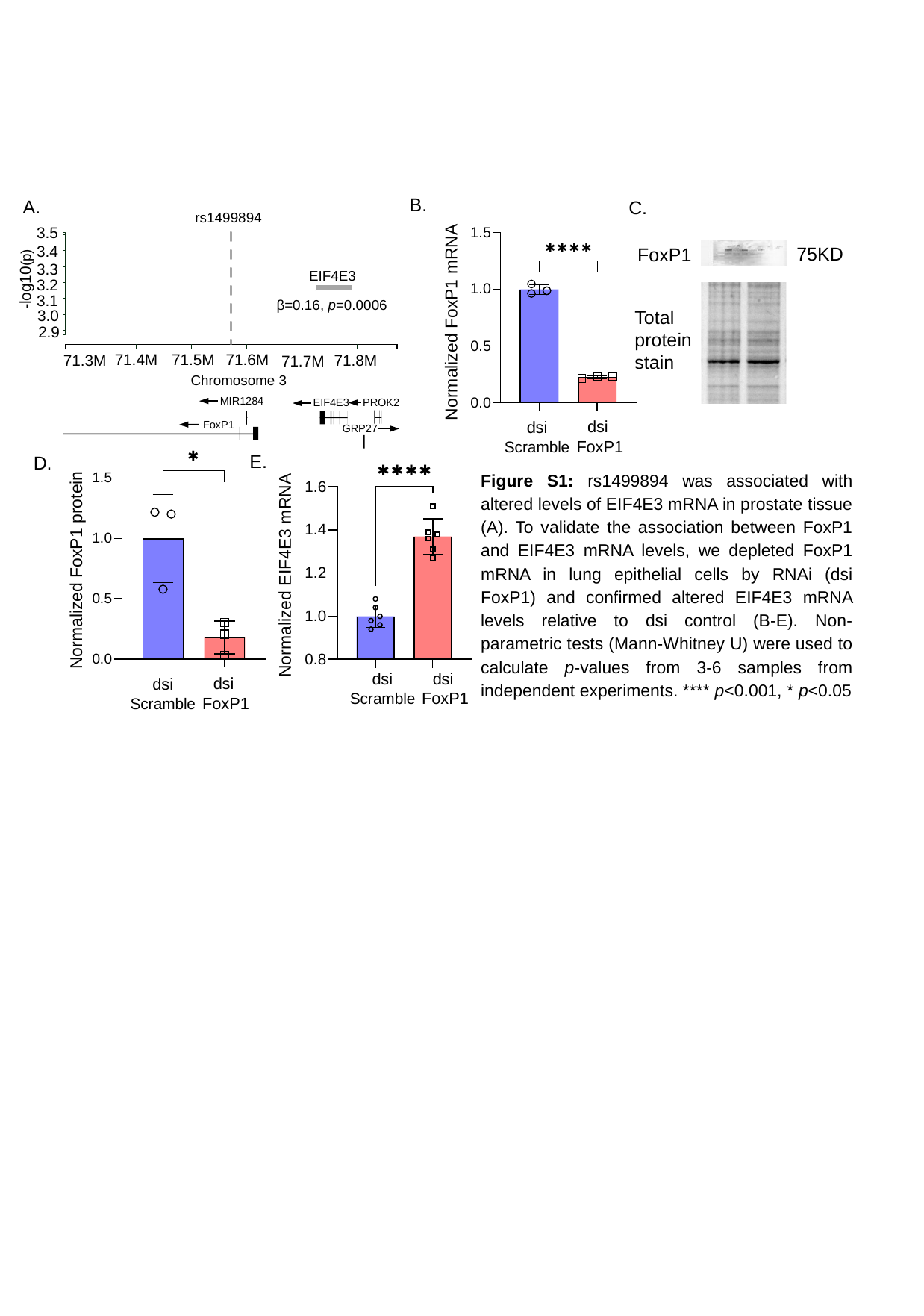

B.
A.
C.
rs1499894
3.5
3.4
-log10(p)
3.3
EIF4E3
3.2
3.1
3.0
2.9
71.5M
71.4M
71.6M
71.3M
71.8M
71.7M
Chromosome 3
MIR1284
EIF4E3
PROK2
GRP27
FoxP1
75KD
FoxP1
β=0.16, p=0.0006
Total
protein
stain
Normalized FoxP1 mRNA
dsi
FoxP1
dsi Scramble
dsi
FoxP1
dsi Scramble
Normalized FoxP1 protein
dsi
FoxP1
dsi Scramble
Normalized EIF4E3 mRNA
E.
D.
Figure S1: rs1499894 was associated with altered levels of EIF4E3 mRNA in prostate tissue (A). To validate the association between FoxP1 and EIF4E3 mRNA levels, we depleted FoxP1 mRNA in lung epithelial cells by RNAi (dsi FoxP1) and confirmed altered EIF4E3 mRNA levels relative to dsi control (B-E). Non-parametric tests (Mann-Whitney U) were used to calculate p-values from 3-6 samples from independent experiments. **** p<0.001, * p<0.05

## Slide 2
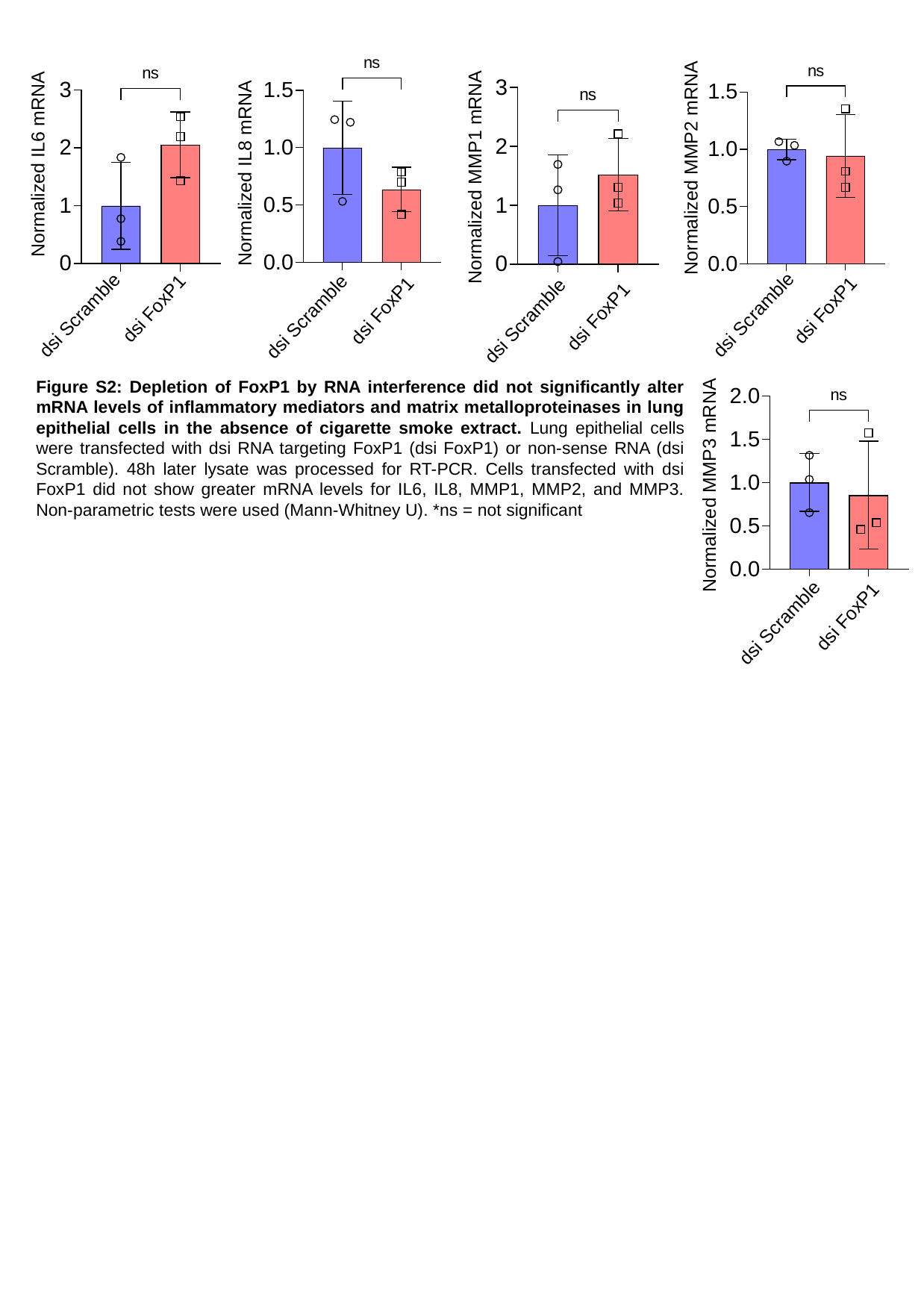

Normalized IL6 mRNA
dsi FoxP1
dsi Scramble
Normalized MMP2 mRNA
dsi FoxP1
dsi Scramble
Normalized IL8 mRNA
dsi FoxP1
dsi Scramble
Normalized MMP1 mRNA
dsi FoxP1
dsi Scramble
Normalized MMP3 mRNA
dsi FoxP1
dsi Scramble
Figure S2: Depletion of FoxP1 by RNA interference did not significantly alter mRNA levels of inflammatory mediators and matrix metalloproteinases in lung epithelial cells in the absence of cigarette smoke extract. Lung epithelial cells were transfected with dsi RNA targeting FoxP1 (dsi FoxP1) or non-sense RNA (dsi Scramble). 48h later lysate was processed for RT-PCR. Cells transfected with dsi FoxP1 did not show greater mRNA levels for IL6, IL8, MMP1, MMP2, and MMP3. Non-parametric tests were used (Mann-Whitney U). *ns = not significant

## Slide 3
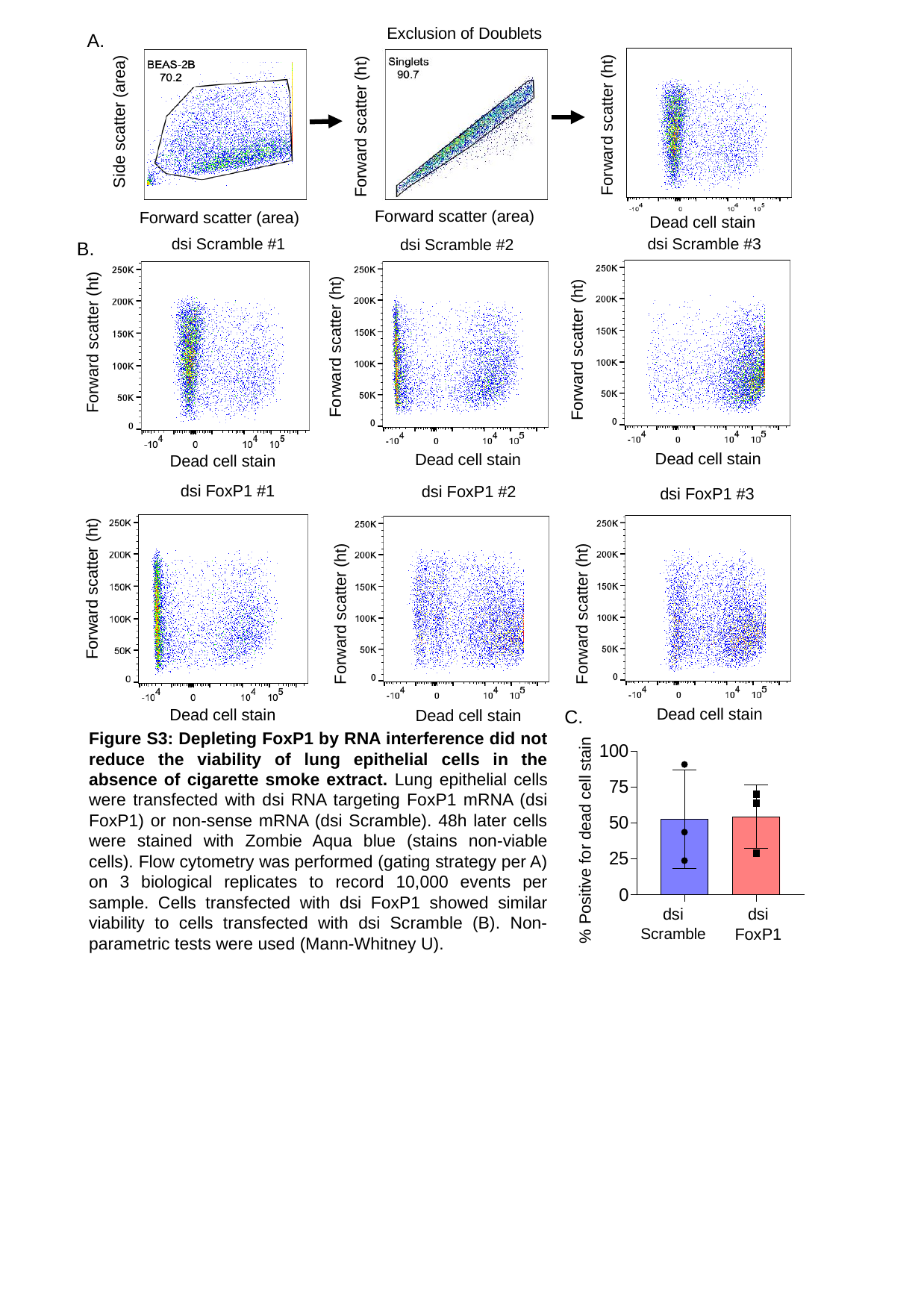

Exclusion of Doublets
A.
Side scatter (area)
Forward scatter (ht)
Forward scatter (ht)
Forward scatter (area)
Forward scatter (area)
Dead cell stain
dsi Scramble #3
dsi Scramble #1
dsi Scramble #2
B.
Forward scatter (ht)
Forward scatter (ht)
Forward scatter (ht)
Dead cell stain
Dead cell stain
Dead cell stain
dsi FoxP1 #1
dsi FoxP1 #2
dsi FoxP1 #3
Forward scatter (ht)
Forward scatter (ht)
Forward scatter (ht)
C.
Dead cell stain
Dead cell stain
Dead cell stain
% Positive for dead cell stain
dsi Scramble
dsi
FoxP1
Figure S3: Depleting FoxP1 by RNA interference did not reduce the viability of lung epithelial cells in the absence of cigarette smoke extract. Lung epithelial cells were transfected with dsi RNA targeting FoxP1 mRNA (dsi FoxP1) or non-sense mRNA (dsi Scramble). 48h later cells were stained with Zombie Aqua blue (stains non-viable cells). Flow cytometry was performed (gating strategy per A) on 3 biological replicates to record 10,000 events per sample. Cells transfected with dsi FoxP1 showed similar viability to cells transfected with dsi Scramble (B). Non-parametric tests were used (Mann-Whitney U).
